# Achievements of malaria elimination program in the face of a difficult situation of population movement with appropriate use of available resources, involvements of research entities and academia

**DOI:** 10.1186/1475-2875-13-S1-P75

**Published:** 2014-09-22

**Authors:** Ahmad Raeisi, Vahid Mirkhani, Fatemeh Nikpour Alkaran, Bita Paktinat Jalali, Leila Faraji, Mansour Ranjbar Keykhah, Seyed Mahdi Tabatabie

**Affiliations:** 1 National Program for Malaria Control/Elimination, CDC, MOH&ME, Tehran, Iran; 2 Department of Medical Entomology & Vector Control, School of Public Health, Tehran University of-Medical Sciences, Tehran, Iran; 3 Zahedan University of Medical Sciences, Zahedan, Iran

## Background

Malaria elimination is one of the important health priorities for the government of the I.R. of Iran. In 2010, the National Strategic Plan for Malaria Elimination was ratified by the High Council for Health and the Governor Generals of three malarious provinces (i.e. Sistan & Balouchestan, Hormozgan and Kerman) were assigned as the core persons for leading the program, dealing with potential challenges, administrative arrangements and financial support. The number of malaria reported cases were 11,000.

## Material and methods

In this survey, malaria surveillance data including weekly, monthly, and web based university reports during the last five years (2009-2013) along with administrative documents related to the malaria program were investigated. In addition, all malaria related research projects and proposals from 2010 to 2014 were reviewed.

## Findings

Given the fact that the malaria elimination program has received strong political commitment from the government, and also academic members and national research centers have been involved in the program, the country has been witnessing tremendous success in tackling malaria across the country. The majority of research topics have shifted to the applied proposals with direct involvement of provincial and district malaria focal points. Moreover, insecticide resistance management, molecular/microscopic monitoring of efficacy of antimalarial and expansion of foci approach to the remote areas are main achievements of this intersectoral collaboration. The great achievements, so far, include: Kerman province now is free from *falciparum* malaria, reducing local malaria cases to 442 at the country level and reduction of active and residual active foci from 1040 to 369. Conclusions Political will has had a strong role in accelerating malaria elimination at the country level. Mutual collaboration of research centers and academia with provincial/district health authorities also has had a crucial role in shifting basic research to the targeted malaria applied projects in line with national priorities.

